# Hyperpolarized Xenon-129: A New Tool to Assess Pulmonary Physiology in Patients with Pulmonary Fibrosis

**DOI:** 10.3390/biomedicines11061533

**Published:** 2023-05-25

**Authors:** Kun Qing, Talissa A. Altes, John P. Mugler, Jaime F. Mata, Nicholas J. Tustison, Kai Ruppert, Juliana Bueno, Lucia Flors, Yun M. Shim, Li Zhao, Joanne Cassani, William G. Teague, John S. Kim, Zhixing Wang, Iulian C. Ruset, F. William Hersman, Borna Mehrad

**Affiliations:** 1 Department of Radiation Oncology, City of Hope National Medical Center, Duarte, CA 91010, USA; zhiwang@coh.org; 2 Department of Radiology, University of Missouri, Columbia, MO 65211, USA; altes@email.chop.edu (T.A.A.); cassanij@health.missouri.edu (J.C.); 3 Department of Radiology and Medical Imaging, University of Virginia, Charlottesville, VA 22903, USA; jpm7r@virginia.edu (J.P.M.III); jfm4q@virginia.edu (J.F.M.); njt4n@virginia.edu (N.J.T.); jmb3dt@hscmail.mcc.virginia.edu (J.B.); yss6n@virginia.edu (Y.M.S.); wgt2p@hscmail.mcc.virginia.edu (W.G.T.); jk6jb@hscmail.mcc.virginia.edu (J.S.K.); 4 Department of Radiology, University of Pennsylvania, Cincinnati, PA 19104, USA; kai.ruppert@gmail.com; 5 Department of Radiology, Keck Medical Center, University of Southern California, Los Angeles, CA 90089, USA; florsbla@usc.edu; 6 Department of Biomedical Engineering, Zhejiang University, Hangzhou 310027, China; lz5bf@virginia.edu; 7 Xemed LLC, Durham, NH 03824, USA; iulian.ruset@xemed.com (I.C.R.); hersman@xemed.com (F.W.H.); 8 Department of Physics, University of New Hampshire, Durham, NH 03824, USA; 9 Department of Medicine, University of Florida, Gainesville, FL 32611, USA; borna.mehrad@medicine.ufl.edu

**Keywords:** hyperpolarized xenon-129 MRI, pulmonary fibrosis, usual interstitial pneumonia

## Abstract

Purpose: The existing tools to quantify lung function in interstitial lung diseases have significant limitations. Lung MRI imaging using inhaled hyperpolarized xenon-129 gas (^129^Xe) as a contrast agent is a new technology for measuring regional lung physiology. We sought to assess the utility of the ^129^Xe MRI in detecting impaired lung physiology in usual interstitial pneumonia (UIP). Materials and methods: After institutional review board approval and informed consent and in compliance with HIPAA regulations, we performed chest CT, pulmonary function tests (PFTs), and ^129^Xe MRI in 10 UIP subjects and 10 healthy controls. Results: The ^129^Xe MRI detected highly heterogeneous abnormalities within individual UIP subjects as compared to controls. Subjects with UIP had markedly impaired ventilation (ventilation defect fraction: UIP: 30 ± 9%; healthy: 21 ± 9%; *p* = 0.026), a greater amount of ^129^Xe dissolved in the lung interstitium (tissue-to-gas ratio: UIP: 1.45 ± 0.35%; healthy: 1.10 ± 0.17%; *p* = 0.014), and impaired ^129^Xe diffusion into the blood (RBC-to-tissue ratio: UIP: 0.20 ± 0.06; healthy: 0.28 ± 0.05; *p* = 0.004). Most MRI variables had no correlation with the CT and PFT measurements. The elevated level of ^129^Xe dissolved in the lung interstitium, in particular, was detectable even in subjects with normal or mildly impaired PFTs, suggesting that this measurement may represent a new method for detecting early fibrosis. Conclusion: The hyperpolarized ^129^Xe MRI was highly sensitive to regional functional changes in subjects with UIP and may represent a new tool for understanding the pathophysiology, monitoring the progression, and assessing the effectiveness of treatment in UIP.

## 1. Introduction

Interstitial lung diseases (ILDs) are diseases characterized by inflammation and fibrosis. Usual interstitial pneumonia (UIP), the most common histologic pattern among ILDs, can result from identifiable causes, including autoimmune diseases and hypersensitivity pneumonitis [1,2]. Additionally, idiopathic pulmonary fibrosis (IPF) is defined as UIP without an identifiable cause [3]. UIP has a poor prognosis [1,4], but its course is difficult to predict in individual patients [5,6]. The identification of patients at risk for rapid deterioration is an important priority, but it cannot be achieved using the current tools. Nindetanib and pirfenidone [7,8] reduce the rate of progression in lung function decline and may improve mortality in IPF [9], but it is not known whether they are more or less effective in specific subsets of patients. Thus, new methods are needed to identify ILD patients who can be targeted for individualized therapy and to monitor treatment effectiveness.

The current tools used in ILDs have notable limitations: pulmonary function testing (PFT) measures only whole-lung physiology—a significant limitation in ILD, in which pathology is heterogeneously distributed. A high-resolution chest CT provides excellent morphologic data on the correlates of inflammation and fibrosis but limited physiologic data. Finally, the histopathology of lung biopsies is limited by sampling bias, weak relationship to physiology, and the infeasibility of obtaining serial samples. Magnetic resonance imaging using hyperpolarized ^129^Xe as a gaseous contrast agent (^129^Xe MRI) is a candidate for the regional quantification of gas-exchange physiology: given its solubility in tissues, ^129^Xe distributes in the lung airspaces and dissolves in the parenchyma and blood in lung capillaries. The dissolution of ^129^Xe in tissues is accompanied by shifts in its resonance frequency, allowing the separate detection of gas-phase, tissue-phase, and RBC-associated ^129^Xe [10,11,12,13,14], providing regional data on lung ventilation, diffusion, and perfusion simultaneously in a single short breath hold [12,15,16,17,18,19].

The quantification of dissolved ^129^Xe can provide data on gas-exchange derangements in lung disease. In animal studies, lung fibrosis results in a reduction in the amount of ^129^Xe reaching the RBCs in the pulmonary circulation [20], and in human interstitial lung disease, MR spectroscopy shows a similar decrease in gas transfer to the blood accompanied by an increase in the amount of ^129^Xe gas dissolved in the lung as compared to healthy volunteers [9]. Mata et al. [21] utilized a three-dimensional (3D) spectroscopic imaging method and demonstrated an increase in ^129^Xe gas uptake in the lung interstitium and a decrease in gas transfer from the interstitium to the blood in 12 patients with IPF as compared to 9 healthy volunteers. This result is consistent with the previous findings by Wang et al. [22] using a 3D radial acquisition and one-point Dixon method [23]. More recently, Hahn et al. used chest CT and a hyperpolarized xenon MRI to try to determine which IPF patients were at greater risk of rapid progression in a cohort of 22 patients with IPF evaluated twice 1 year apart [24]. At 1 year, there were 9 progressors and 13 nonprogressors in the IPF cohort, and in this small cohort, a relative decrease in RBC gas exchange at baseline was associated with progression at 1 year.

The 3D spectroscopic imaging method has limited spatial resolution due to its long acquisition time for each spectrum. The accuracy of the one-point Dixon method relies on a special timing for the alignment of the tissue-phase and RBC-associated ^129^Xe signal in the real and imaginary channels of the MRI signal receiver. This special timing is determined based on a global spectrum measurement and may vary to a certain extent among imaging voxels with different composition and field inhomogeneities. Our group developed a hyperpolarized ^129^Xe MRI technique [25] based on a three-point Dixon method that provides 3D regional information on the ventilation and gas uptake in the human lung. A typical three-point Dixon–based method provides more reliable separation of the tissue-phase and RBC-associated ^129^Xe signal, especially in diseased patients with more heterogeneous distributions of the ^129^Xe gas uptake signal.

The purpose of the current study was to test the ability of our technique to detect changes in lung physiology in patients with pulmonary fibrosis as compared to healthy controls and to compare lung ^129^Xe MRI results with conventional clinical indices of disease severity, namely, pulmonary function tests and high-resolution chest CT imaging.

## 2. Materials and Methods

### 2.1. Subjects

The study was performed under an Investigational New Drug application from the United States Food and Drug Administration for ^129^Xe MRI, using a protocol approved by the Institutional Review Board and in compliance with HIPAA. Written informed consent was obtained from all subjects. We prospectively recruited 10 healthy control subjects and 10 subjects with UIP in a case–control study between September 2012 and November 2015. The controls were recruited by advertisements and were never-smokers with no history of lung disease. The UIP subjects were recruited consecutively from the University of Virginia Interstitial Lung Disease clinic and were identified on the basis of the histopathologic examination of surgical lung biopsy samples or diagnostic patterns on high-resolution chest CT, according to consensus guidelines [3]. For UIP subjects, the final clinical diagnoses were reached after a multidisciplinary review by a group consisting of interstitial lung disease specialists, thoracic radiologists, and a pulmonary pathologist. The repeatability of the ^129^Xe MRI studies was tested in 4 healthy controls and on all UIP subjects on the same day.

All subjects underwent pulmonary function tests including spirometry and diffusion capacity for carbon monoxide, and the tests were interpreted according to the published criteria [9]. High-resolution CT scans were acquired for each subject. The parameters for the CT protocol were as follows: kVP = 120, mA = 35, mAs = 130–220, pitch = 1.2, slice thickness = 1.2 mm, matrix = 512 × 512, radiation dose = 3–8 mSv. The CT data were retrospectively reviewed and scored independently and in random order by 2 chest radiologists (J.B. and L.F.; each with >10 years of experience), who were blind to the clinical data. Using a validated visual scoring system, the mean extent of the reticular abnormality and honeycombing were scored to the nearest 5% in 3 zones in each lung (at or above the aortic arch, between the aortic arch and the pulmonary veins, and at or below the pulmonary veins) [26]. The scores for total lung fibrosis were calculated as the sum of the scores of reticular abnormality and honeycombing.

### 2.2. MR Acquisition and Data Processing

MRI acquisitions were performed using a 1.5T commercial whole-body scanner (Avanto; Siemens Medical Solutions, Malvern, PA, USA) and a vest-shaped chest RF coil (Clinical MR Solutions, Brookfield, WI, USA). Enriched xenon gas (87% ^129^Xe) was used and polarized by a prototype commercial system (XeBox-E10; Xemed, LLC, Durham, NH, USA). Since patients with interstitial lung disease have variably reduced lung volumes as compared to normal subjects and the measurements are dependent on the quantity of the inhaled hyperpolarized ^129^Xe, we standardized the volume of inhaled gas to each subject’s lung volume: each subject inhaled a volume of hyperpolarized ^129^Xe and nitrogen equal to one-third of their forced vital capacity (FVC, based on spirometry obtained immediately prior to imaging). Inhalations of the hyperpolarized ^129^Xe gas were from maximum exhalation, followed by a 10 s breath hold for the duration of the imaging acquisition. Ventilation data were acquired using multislice, 2D combined imaging of ^129^Xe ventilation and proton anatomy (ventilation: duration = 3 s, TR = 11.4 ms, TE = 1.19 ms, field of view = 50 cm, matrix size = 128 × 128, 15 slices, resolution 3.9 × 3.9 × 15 mm^3^, spiral readout with 12 interleaves, flip angle = 20°; proton: duration = 2 s, TR = 4.8 ms, TE = 0.9 ms, field of view = 50 cm, matrix size = 128 × 128, 15 slices, resolution 3.9 × 3.9 × 15 mm^3^, spiral readout with 22 interleaves, flip angle = 15°), and dissolved-phase data were acquired in 3D (duration 11 s, and resolution 7.6 × 7.6 × 17.6 mm^3^, radial readout, TR = 19 ms, TE1/TE2/TE3 = 0.74/2.36/3.98 ms for the dissolved-phase ^129^Xe and TE1/TE2 = 0.74/2.36 ms for the gas-phase ^129^Xe, flip angle = 23° for the dissolved-phase ^129^Xe and 0.4° for the gas-phase ^129^Xe).

An automated segmentation method [27] was used to quantify ventilation based on the combined ^129^Xe and proton acquisition images. Lung regions were segmented into 4 classes based on signal intensity, corresponding to nonventilated, hypoventilated, ventilated, and well-ventilated areas. The percentage of the lung with reduced ventilation (V_def_) was calculated as the sum of the nonventilated and hypoventilated volumes normalized to the total lung volume. Image registration and segmentation were processed using the Advanced Normalization Tools package [28].

The ^129^Xe dissolved-phase images were processed as previously described [25]. Data were expressed as the tissue-to-gas ratio (defined as the percent of ^129^Xe dissolved in tissue compared to that in the airspaces) reflecting the ^129^Xe bound in the interstitial tissues and the RBC-to-tissue ratio (ratio of ^129^Xe dissolved in RBC to that in tissue) reflecting the efficiency of gas transfer from tissue to the RBCs in pulmonary circulation.

### 2.3. Data Analysis

Statistical analyses were performed in MATLAB (Mathworks, Natick, MA, USA). The Mann–Whitney U test was used to evaluate the difference in lung function measures between groups. A two-tailed Pearson correlation was used to evaluate the relationships among the ^129^Xe imaging variables, PFT variables, and CT scores. The interreader agreement on CT scores was assessed using the Krippendorff’s alpha coefficient for ordinal data. Probability values of less than 0.05 were regarded as statistically significant.

## 3. Results

### 3.1. Subject Demographics and PFTs

The characteristics of UIP and the control subjects are summarized in Table 1. The PFTs were normal in all control subjects. Individual UIP subject data are listed in the supplemental table (online material). Among the UIP subjects, seven had IPF, and UIP was secondary to connective tissue disease in two subjects and to drug toxicity in one subject. UIP was identified based on histopathology in six subjects and CT in four subjects. As expected, subjects with UIP had significantly lower forced vital capacity and gas transfer as compared to the healthy controls.

### 3.2. ^129^Xe MRI Quantification of Ventilation in UIP

**Figure 1:**
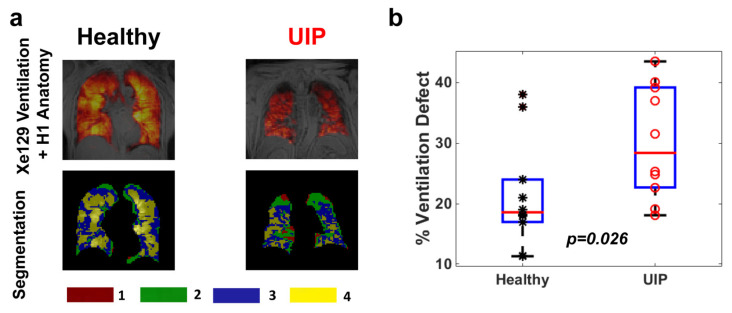
Panel (**a**): Upper: representative combined imaging of ^129^Xe ventilation and proton anatomy from one healthy volunteer and a subject with UIP. Lower: corresponding segmentation maps. Regions labeled as 1 represent nonventilated lung areas and regions labeled 2–4 represent areas with increasing ventilation. Panel (**b**): Ventilation defects in 10 healthy subjects (marked as *) and 10 patients with UIP (marked as °). Hypoventilated areas were defined as regions 1 and 2 in Panel (**a**); the percent ventilation defect was calculated as the sum of regions 1 and 2 divided by the total lung volume.

All subjects were able to complete the ^129^Xe gas inhalation maneuver. We found a marked degree of ventilation defect in subjects with UIP. The upper panels of Figure 1a show representative ^129^Xe ventilation images overlaid with proton anatomy images, both acquired in a single breath hold, from a control subject and a subject with UIP. Using an automated segmentation method to quantify ventilation, the lung segments were categorized as nonventilated, hypoventilated, ventilated, and well-ventilated areas based on signal intensity; the percentage of the total lung volume with reduced ventilation (V_def_) was also calculated. The lower panels of Figure 1a show the ventilation-based segmentation maps corresponding to the upper panels. As a group, the subjects with UIP had significantly more ventilation defects as compared to the control group (30% vs. 21%, *p* = 0.026, Figure 1b).

### 3.3. Measures from Dissolved-Phase ^129^Xe MRI and Comparison with CT

**Figure 2:**
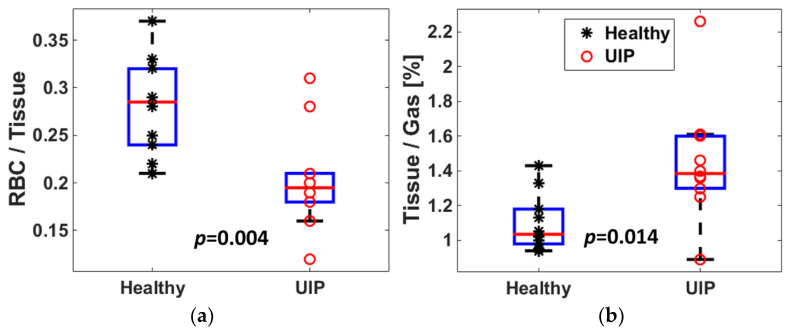
Whole-lung tissue-to-gas (Panel (**a**)) and RBC-to-tissue (Panel (**b**)) ratios in 10 healthy and 10 UIP subjects who underwent dissolved-phase ^129^Xe MRI.

**Figure 3:**
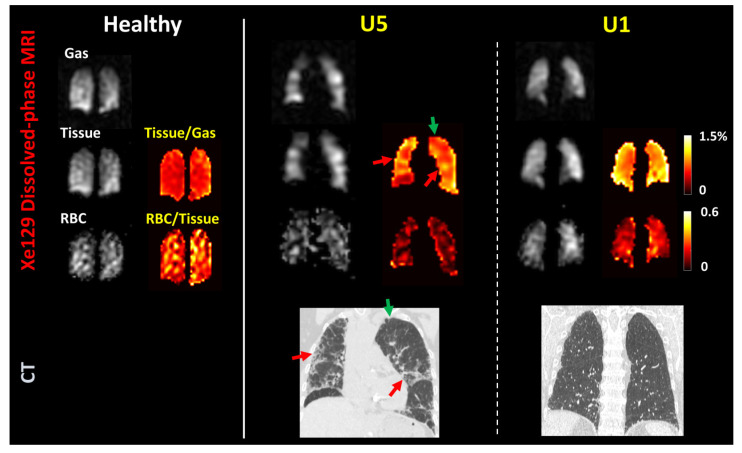
Representative dissolved-phase ^129^Xe MRI (**top**) and coronal high-resolution CT (**bottom**) results from one healthy subject (52-year-old woman, FVC 113% predicted, DLCO 111% predicted) and two subjects with UIP (subject U5 in the supplemental table: 79-year-old woman, FVC 53% predicted, DLCO 31% predicted; subject U1 in the supplemental table: 73-year-old man, FVC 76% predicted, DLCO 61% predicted). “Gas,” ^129^Xe in the airspace; “Tissue,” ^129^Xe dissolved in the lung interstitium; “RBC,” ^129^Xe associated with red blood cells. “Tissue/gas” and “RBC/Tissue” panels show the calculated tissue-to-gas and RBC-to-tissue ratio maps for each subject. Red arrows show areas of more severe fibrosis on CT corresponding to areas of increased tissue-to-gas ratios, and green arrow indicate areas of relatively normal lung on CT corresponding to areas of close to normal tissue-to-gas ratios.

The CT and MRI findings of UIP subjects are summarized in Table 2; the CT scores between readers showed excellent agreement (Krippendorff′s alpha 0.94). We assessed the absorption of hyperpolarized ^129^Xe from the airspaces into the interstitium by quantifying the ratio of ^129^Xe content in the lung parenchyma to that in the gas phase; similarly, we assessed the uptake of gas into the blood by measuring the ratio of the ^129^Xe content in the red blood cells within pulmonary circulation to that in lung parenchyma. We found the ^129^Xe MRI tissue-to-gas ratios to be significantly higher in subjects with UIP as compared to controls (Figure 2a and Table 2), whereas the RBC-to-tissue ratio was significantly lower (Figure 2b and Table 2). Figure 3 shows representative ^129^Xe dissolved-phase images and ratio maps (upper panels) from a healthy control subject, a UIP subject with severe physiologic impairment, and another UIP subject with preserved lung function (subjects U5 and U1 from Supplementary Table S1, respectively), together with CT images of the UIP subjects at similar coronal planes. As compared to the control subjects, the subject with severe disease had a low RBC-to-tissue ratio and a highly heterogeneous tissue-to-gas ratio, with a high whole-lung mean tissue-to-gas ratio. Areas of more severe fibrosis on CT corresponded to areas of increased tissue-to-gas ratios (indicated by red arrows) and areas of relatively normal lung on CT corresponded to areas of close to normal tissue-to-gas ratios (indicated by green arrow), supporting the contention that the elevated tissue-to-gas ratio is a measure of the extracellular matrix in UIP subjects. The subject with less severe disease had only minor reticulation on CT and normal or mildly impaired PFT but significantly elevated tissue-to-gas ratios (whole-lung mean 1.61%) and also low RBC-to-tissue ratios (whole-lung mean 0.16), especially in the periphery of the lungs.

**Figure 4:**
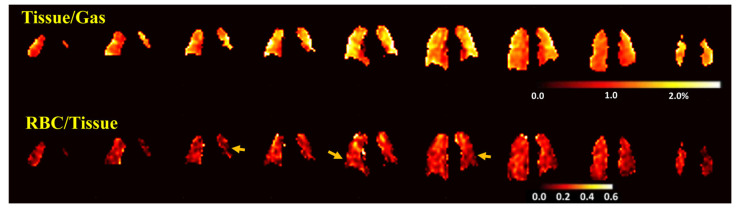
Tissue-to-gas and RBC-to-tissue ratio maps covering the entire lung in a subject with UIP (subject U2 in the supplemental table, 70-year-old man) showing heterogeneous distributions of ^129^Xe, with elevated tissue-to-gas ratios and low RBC-to-tissue ratios in the periphery of the lungs (arrows).

The intersubject heterogeneity of the tissue-to-gas ratios was large, with ratios ranging from 0.89% to 2.26% (Figure 2a). Furthermore, the tissue-to-gas ratio maps were highly heterogeneous within each UIP subject, with the highest values in the periphery of the lungs, corresponding to areas of reticulation and honeycombing on CT, while the RBC-to-tissue ratios were lower in areas with increased tissue-to-gas ratios, again suggesting that the higher tissue-to-gas ratio reflects extracellular matrix deposition. As an example of this, Figure 4 shows the tissue-to-gas and RBC-to-tissue ratio maps covering the entire lung from a UIP subject (subject U2 from Supplementary Table S1). Much of the peripheral and basal lungs had higher tissue-to-gas ratios (bright yellow) than the central regions of the lungs, corresponding to areas of lung with more severe fibrosis. Within these areas, the RBC-to-tissue ratios were lower (darker red areas indicated by arrows) than other areas of the lungs, corresponding to impaired gas uptake into the blood in areas of more severe fibrosis.

### 3.4. Repeatability of ^129^Xe Ventilation and Dissolved-Phase MRI Data

**Figure 5:**
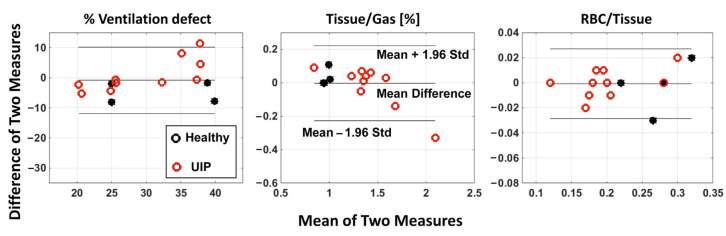
Bland–Altman plots of the total ventilation defect volume, tissue-to-gas ratio, and RBC-to-tissue ratio measured by repeated ^129^Xe ventilation and dissolved-phase MRI acquisitions. Horizontal lines represent mean and 95% confidence intervals.

All ^129^Xe MRI variables were found to be highly repeatable, with a variability in whole-lung measurements of V_def_, tissue-to-gas ratio, and RBC-to-tissue ratio of 6.5%, 5% and 4%, respectively. We also assessed the repeatability of these measures using the Bland–Altman method (Figure 5). The mean differences between the repeated measurements were found to be −0.85 ± 5.51% for V_def_, −0.004 ± 0.114% for the tissue-to-gas ratio, and −0.001 ± 0.014% for the RBC-to-tissue ratio.

### 3.5. Correlations between ^129^Xe MRI, PFTs, and CT Variables in UIP Subjects

**Figure 6:**
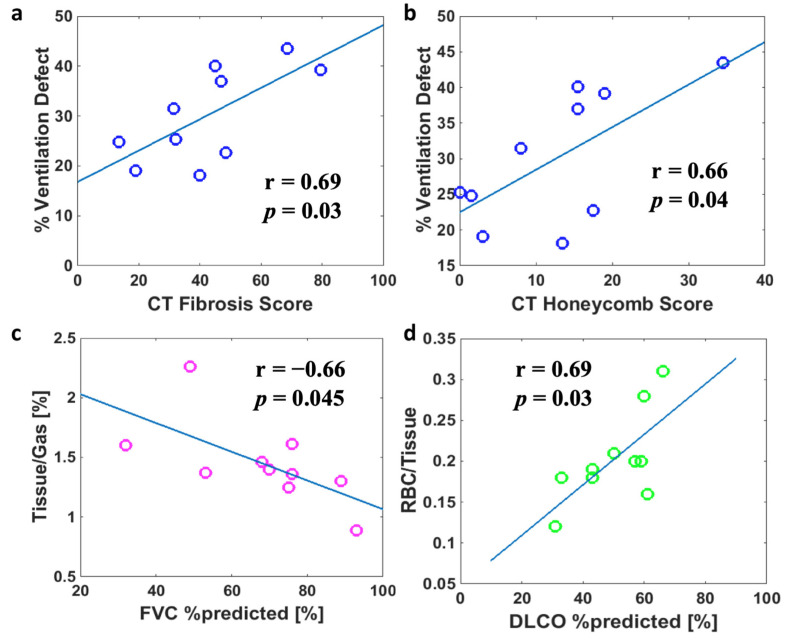
Statistically significant correlations between ^129^Xe MRI variables and CT and PFT variables in subjects with UIP. Individual subjects are represented by circles and lines show linear regression results.

Lastly, we assessed the correlations both within the ^129^Xe MRI measurements and between the ^129^Xe MRI variables and conventional clinical measures of lung function, namely, PFTs and chest CT (Table 3). The V_def_ did not correlate significantly with any PFT or dissolved-phase ^129^Xe measures but correlated with the scores for honeycombing and total fibrosis on CT (Figure 6a,b), suggesting that the areas of impaired ventilation noted in UIP subjects (Figure 1) may be attributable to reduced ventilation to areas of fibrosis/scarring. Only two correlations reached statistical significance for the ^129^Xe dissolved-phase measurements: As expected, there was a significant correlation between DLCO and RBC-to-tissue ratio (Figure 6c), consistent with the notion that both variables quantify gas absorption into the bloodstream. In addition, the FVC and tissue-to-gas ratio had a significant negative correlation (Figure 6d). This finding supports the hypothesis that the tissue-to-gas ratio may represent a new measure of tissue fibrosis.

## 4. Discussion

The identification of biomarkers for disease activity, therapeutic responsiveness, and prognosis is a priority in interstitial lung diseases [29]. In contrast to the emerging understanding of the cellular and molecular pathobiology of lung fibrosis, the clinical tools for the measurement of lung physiology in interstitial lung diseases provide only whole-organism data and correlate poorly with disease progression. The new technology of hyperpolarized ^129^Xe MRI has the potential to fill this gap by providing detailed regional data on gas exchange.

Subjects with UIP had significantly lower RBC-to-tissue ratios as compared to control subjects, and RBC-to-tissue ratios correlated with DLCO, confirming that this value quantifies gas diffusion from the airspaces to blood. These results are consistent with findings by other researchers [21,22,24,30,31]. For example, Wang et al. [22] found an increase in barrier uptake (tissue-to-gas ratios) by 188% in 12 older patients (66.0 ± 6.4 yrs) with IPF as compared to a younger healthy volunteer group (33.6 ± 5.7 yrs). The RBC-to-tissue transfer in [22] was low in several patients and correlated strongly with DLCO (r = 0.94). Compared to Wang et al. [22], our healthy volunteers were more age-matched to the patient group. Similarly, Kaushik SS et al. [32] and Mata et al. [21] and found significantly higher tissue-to-gas ratios and lower RBC-to-gas ratios by using spectroscopy or spectroscopic imaging. More interestingly, Mata et al. [21] discovered a lower chemical shift in the RBC spectroscopic peak, which may be explained by reduced blood oxygenation. A decrease in the RBC-to-tissue ratio has previously been shown to reflect impairment of diffusion or reduced pulmonary perfusion in pre-clinical animal models [16,17]. We also found significant elevations in the tissue-to-gas ratio that were negatively correlated with FVC, consistent with Wang et al. [22]. This finding stands in contrast to the ^129^Xe MRI findings for chronic obstructive pulmonary disease, where factors such as hyperexpansion and vascular pruning were associated with the decreased tissue-to-gas ratio detected by ^129^Xe MRI [33]. Given that increased extracellular matrix in interstitial lung diseases is related to lung restriction as measured by FVC, the tissue-to-gas ratio from ^129^Xe MRI may represent a more sensitive measure of the extent of extracellular matrix deposition in these conditions. Furthermore, the coexistence of high tissue-to-gas and low RBC-to-tissue ratios in UIP patients, for example as shown in Figure 4, provides a potential physiologic link between the extracellular matrix volume and the mechanisms of hypoxia in UIP. Intriguingly, some subjects with UIP with relatively mild changes on CT still had high tissue-to-gas ratios. A possible explanation of this finding could be that the functional alterations detected by ^129^Xe MRI temporally precede the morphological changes detected by CT; ^129^Xe MRI may, therefore, be more sensitive to early changes in UIP. This hypothesis awaits confirmation in longitudinal studies.

Another key finding was the high degree of heterogeneity in the distribution of gas transfer in UIP. A key radiological feature of UIP on CT is the presence of lesions along the basal and sub-pleural regions [9]. We observed similar high tissue-to-gas ratios at these locations in the ratio maps, as shown in Figure 4. These are similar to the “stronger central–peripheral gradient” observed by Wang et al. [22]. Furthermore, the RBC-to-tissue ratios detected in areas with high tissue-to-gas ratios appeared to be low as compared to other regions, indicating impaired ^129^Xe diffusion into the blood in areas with fibrosis. In this context, the impairment of diffusion is found to precede changes in lung volume measured by PFTs in patients with early-stage IPF [34]. The efficiency of diffusion processes, however, can vary widely within the lung in heterogeneous diseases such as IPF. The ^129^Xe MRI, with the ability to provide regional information on the lung function, could be more sensitive to detect these early changes in pathophysiology.

An unexpected finding in this study was a notable increase in areas of hypoventilation in subjects with UIP as compared to healthy controls, which was not observed by Wang et al. [22]. Interestingly, impaired ventilation did not correlate with PFT variables or with dissolved-phase ^129^Xe MRI measures but was significantly associated with the honeycombing and fibrosis scores on CT. The mechanisms and significance of ventilation defects in UIP remain to be defined in future studies.

We recognize a number of limitations to this study. The relatively small number of subjects in this pilot study limits the ability to generalize or apply findings to all subjects with UIP, but the excellent repeatability of the ^129^Xe MRI variables provides a strong basis for future studies on this modality. In addition, although we found no relationship between age, sex, and ^129^Xe MRI measurements, the small sample size precludes the detection of such trends. Similarly, the small sample size may have precluded the detection of correlations between some MRI measurements and CT or PFT variables and the detected correlations may have been stronger with a larger study population. Ours was a cross-sectional study and does not provide longitudinal data on the relevance of the ^129^Xe MRI findings to subsequent clinical events, for example, to assess response to antifibrotic medications. Finally, this is a single-center study, and replication of the findings by other investigators is required for the external validation of ^129^Xe MRI in UIP.

In summary, our study confirms that ^129^Xe MRI is capable of detecting alterations in ventilation and gas exchange in UIP. With previously unobtainable 3D resolution covering the entire lung, we observed that the gas transfer in lungs with UIP was highly heterogeneous, even in patients with mild changes on CT and PFTs. With the recent FDA approval of 129Xe MRI for ventilation imaging in December 2022, there may be an increase in the number of sites able to perform 129Xe MRI. Given the relatively low numbers of UIP/IPF patients, developing mechanisms to facilize the aggregation of multicenter clinical and research data from 129Xe MR in UIP may be of use to gain insights into whether specific patterns on 129Xe MRI identify subsets of patients with different responses to therapy or prognosis.

## Figures and Tables

**Table 1:** Demographics and PFT results in study subjects.

	Healthy Controls	UIP	*p* Value
Number (male/female)	10 (5/5)	10 (7/3)	0.65
Median age (IQR)	57 (50–65)	69 (62–75)	0.15
Median % predicted * FVC (IQR)	108 (98–114)	73 (57–80)	<0.0001
Median % predicted * DLCO (IQR)	94 (83–103)	54 (41–60)	<0.0001

* Predicted PFT values were calculated using NHANES III reference values. DLCO, diffusion capacity for carbon monoxide; FVC, forced vital capacity; IQR, interquartile range; UIP, usual interstitial pneumonia.

**Table 2:** Summary CT and ^129^Xe MRI data in the study subjects.

		Healthy Controls	UIP	*p* Value
HRCT variables	% Reticulation	n/a	31 (22–33)	n/a
	% Honeycomb change	n/a	14 (2.8–20)	n/a
	% Total fibrosis	n/a	43 (29–54)	n/a
^129^Xe MRI variables	% Ventilation defect	19 (15–27)	29 (22–39)	0.022
	Tissue-to-gas ratio	1.0 (0.97–1.2)	1.4 (1.3–1.6)	0.014
	RBC-to-tissue ratio	0.27 (0.22–0.28)	0.20 (0.18–0.23)	0.004

HRCT, high-resolution chest computer tomography; RBC, red blood cell; UIP, usual interstitial pneumonia.

**Table 3:** Correlations between ^129^Xe MRI measures, PFTs, and CT scores.

	Vdef	%FVC	%FEV1	FEV1/FVC	%FEF25–75	%DL	%DL/Va	Relic	HC	Fibrosis
Vdef	-	0.11 (0.77)	0 (0.99)	−0.15 (0.69)	−0.55 (0.10)	−0.10 (0.78)	0.37 (0.29)	0.55 (0.10)	0.66 (0.04)	0.69 (0.03)
Tissue/gas	−0.37 (0.30)	−0.64 (0.045)	−0.61 (0.06)	0.16 (0.66)	0.20 (0.57)	−0.39 (0.26)	0.31 (0.38)	0.07 (0.85)	−0.54 (0.11)	−0.23 (0.52)
RBC/tissue	0.12 (0.74)	0.57 (0.08)	0.46 (0.18)	−0.47 (0.17)	−0.43 (0.21)	0.69 (0.03)	0.29 (0.41)	−0.08 (0.83)	0.03 (0.93)	−0.03 (0.93)

Data are shown as correlation coefficient r (*p* value). Correlations with *p* value < 0.05 are outlined in gray. %DL, percent predicted diffusion capacity for carbon monoxide; %DL/Va, percent predicted diffusion capacity for carbon monoxide corrected for alveolar volume; %FEF_25–75_, percent predicted forced expiratory flow between 25% and 75% of FVC; %FEV_1_, percent predicted forced expiratory volume in 1 s; %FVC, percent predicted forced vital capacity; Fibrosis, mean total lung fibrosis score on CT; HC, mean honeycombing score on CT; Retic, mean reticulation score on CT; V_def_, proportion of lung volume with impaired ventilation.

## Data Availability

The data presented in this study are available on request from the corresponding author.

## Supplementary Materials

The following supporting information can be downloaded at https://www.mdpi.com/article/10.3390/biomedicines11061533/s1: Table S1: Demographics, diagnostic categories, PFT data, and mean CT scores in individual UIP subjects.
