# Supplementary material for: Hyperpolarized Xenon-129: A New Tool to Assess Pulmonary Physiology in Patients with Pulmonary Fibrosis
Source: Biomedicines. 2023 May 25;11(6):1533. doi: 10.3390/biomedicines11061533 (PMC10294784; doi:10.3390/biomedicines11061533)
Supplement: Supplementary file 1 [file biomedicines-11-01533-s001.zip › biomedicines-2366955-supplementary.pdf]

ONLINE SUPPLEMENT FOR

Hyper-polarized xenon-129 magnetic resonance imaging: A new tool to quantify functional derangement in pulmonary fibrosis

Supplementary Table S1. Demographics, diagnostic categories, PFT data and mean CT scores in individual UIP subjects.

Subjects	Age	Sex	Dx	UIP basis	PFT (% predicted)		Chest HRCT		
					FVC	DLCO	Ret	HC	Emph
U1	73	M	IPF	Path	76	61	16	2	
U2	70	M	IPF	Path	70	57	24	7	
U3	80	M	IPF	Path	75	43	34	31	
U4	38	F	DM	Path	32	33	61	29	
U5	79	F	IPF	CT	53	31	31	17	
U6	68	F	IPF	CT	89	59	30	14	
U7	56	M	DM	Path	76	60	27	14	
U8	64	M	drug	Path	49	43	32	0	
U9	72	M	IPF	CT	93	66	12	3	
U10	64	M	IPF	CT	68	50	32	15	

Predicted PFT values were calculated using NHANES III reference values.

DLCO, diffusion capacity for carbon monoxide; Dx, diagnosis; FVC, forced vital capacity; HC, honeycomb change; Ret, reticular opacities. None of the UIP subjects had an obstructive ventilatory defect on PFT or mosaic ground glass attenuation on CT.
